# Polymer–DNA assembled nanoflower for targeted delivery of dolastatin-derived microtubule inhibitors†

**DOI:** 10.1039/d3ra08146j

**Published:** 2024-03-21

**Authors:** Tiantian Wu, Yanqiang Shi, Tao Yang, Pengxuan Zhao, Zhu Yang, Bin Yang

**Affiliations:** a Molecular Diagnosis and Treatment Center for Infectious Diseases, Dermatology Hospital, Southern Medical University Guangzhou 510091 China yangbin1@smu.edu.cn; b Key Laboratory of Tropical Translational Medicine of Ministry of Education, Hainan Provincial Key Laboratory for Research and Development of Tropical Herbs, School of Pharmacy, Hainan Medical University Haikou 571199 China; c Department of Neurosurgery, Neurosurgery Research Institute, The First Affiliated Hospital of Fujian Medical University Fuzhou 350005 China,; d Department of Neurosurgery, National Regional Medical Center, Binhai Campus of the First Affiliated Hospital of Fujian Medical University Fuzhou 350212 China

## Abstract

Dolastatin derivatives possess excellent anticancer activity and have been translated into clinical trials for cancer therapy. Drug delivery systems enable dolastatin derivatives to break the limitation of instability during blood circulation and ineffective cell internalization in the application. Nevertheless, their potential has not been thoroughly established because of the limited loading efficacy and complicated chemical modification. Herein, we rationally propose a rolling circle amplification-based polymer–DNA assembled nanoflower for targeted and efficient delivery of dolastatin-derived drugs to achieve efficient anticancer therapy. The polymer–DNA assembled nanoflower with targeted aptamer conjugate is widely applicable for loading dolastatin-derived drugs with high encapsulation efficiency. The developed monomethyl auristatin E (MMAE) loaded PN@M exhibited increased cellular uptake and enhanced inhibitory effect, especially in multidrug-resistant tumor cells. The results of *in vivo* anticancer effects indicate that nanoflower as a dolastatin derivatives delivery system holds considerable potential for the treatment of malignant cancer.

## Abstract 2

Polymer–DNA assembled nanoflower for targeted delivery of dolastatin-derived microtubule inhibitors was developed, and it exhibited increased cellular uptake and enhanced inhibitory effect, especially in multidrug-resistant tumor cell.

## Graphical abstract

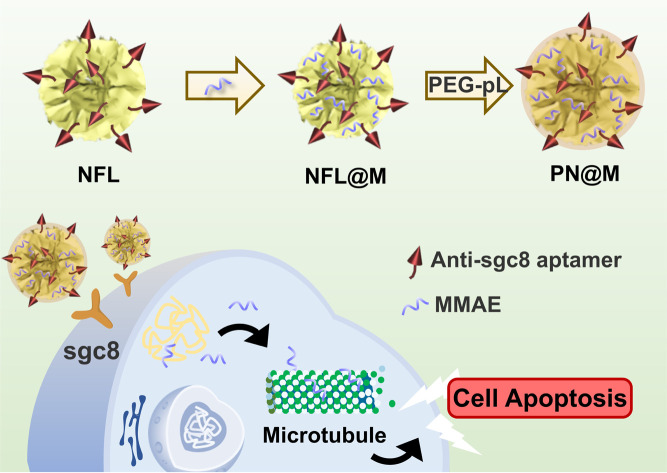

## Introduction

Marine-derived compounds, such as those comprising alkaloids, cyclodepsipeptides, hydroxyphenylacetic acid derivatives, terpenoids, and polyketide, demonstrate a significant specificity and potent affinity for selecting biological targets that are associated with specific intracellular signaling pathways.^1–3^ Compared with the traditional chemical-synthesized small molecule inhibitors, marine-derived compounds inspired by natural active ingredients present higher activity, stronger targeting, and lower toxicity. Marine-derived compounds, especially marine-derived peptides, were broadly studied in biomedical applications such as antitumor, anti-inflammatory, antivirus, and anti-infection therapy. Since the 21st century, the great potential of unique marine natural products as candidate therapeutic agents has been widely recognized by researchers.^4^ Considerable attention from researchers has focused on marine bioactive agents due to their novel chemical and biological properties against cancer cells and significant advancement has been seen in the clinical research of marine-derived medicines applied in malignancy therapy.^5,6^ Dolastatins were initially isolated from the mollusc *Dolabella auricularia*.^7–9^ The excellent anti-tumor effect of the dolastatins was demonstrated and considerable efforts were made to study the mechanisms and possible potentials.^7,10–12^ Anticancer agents such as auristatin PE (PE), dolastatin-10 (D-10), monomethyl auristatin E (MMAE), and monomethyl auristatin F (MMAF) are typical dolastatin derivatives used for antitumor therapy. These derivatives have attracted much attention due to strong anti-cancer activity through tubulin polymerization inhibition. However, MMAE was proved to have limitations as a drug itself. Due to the non-specificity of cell internalization, MMAE holds defects in enriching in tumor through systemic administration and could cause severe systemic toxicity during the treatment.^13–15^ Thus, the necessity of making improvements in the administration of drugs derived from marine natural compounds is universal and garnering increasing interest.

A nano particle-based delivery system has been developed and universally testified to evidently improve drug effects for several advantages:^16–20^ (1) avoiding drug degradation in the physiological environment; (2) maintaining continual drug release to extend the effective medication concentration; and (3) achieving targeted delivery to improve the specificity. Based on these superiorities, a variety of delivery systems were developed for clinical translation for further application.^21^ As reported, a broad diversity of nano particle-based delivery systems, such as liposomes,^21,22^ polymer micelles,^23,24^ and metal nanoparticles^25,26^ have been constructed and advanced the process of natural compound-based drug delivery. However, the limited specificity and potential toxicity of the mentioned delivery system have plagued scientists for decades.^27,28^ Delivery systems with universality, high drug-loading capability, and targetability are essential and rare for dolastatin-derived drugs.

A DNA self-assembly structure with rationally designed geometries and excellent biocompatibility has provided a platform for multifunctional drug delivery.^29–31^ DNA, as a classic endogenous carrier of genetic information, can also be employed as the building block to construct DNA structures by complementary base pair engineering. Precisely self-assembled DNA structures have been widely developed for biomedical applications. A drug delivery system is one of the most studied directions of DNA self-assembly structure in biomedical applications.^32–35^ DNA self-assembly structures include multiple types, such as DNA origami, DNA tile, DNA polyhedron, and DNA dendrimers; each type has a unique performance in drug delivery application. Rolling circle amplification (RCA), an enzymatic process that produces repetitive single-stranded DNA utilizing circular DNA as a template,^36,37^ can be used for synthesizing self-assembly nanoflowers.^38,39^ The feature of the highly tandem repeating sequences of DNA strands in self-assembly nanoflowers can maximize the performance of functional nucleic acids-based active agents. The functional ligands, which play prominent roles in the selective recognition of cells, targeted delivery, and therapeutics are simply equipped in DNA self-assembly nanoflowers through complementary base pairing.^40,41^ Nucleic acid-based functionalities, such as DNA or RNA aptamer for cell targeting, antisenses oligonucleotides for gene therapy, and metal ion-involved DNAzymes for catalytic reaction have been developed to be modified in nanoflowers through rational design.^32,33^ Taking advantage of these features, nanoflowers are extensively studied for the development of multifunctional drug delivery systems.

**Fig. 1:**
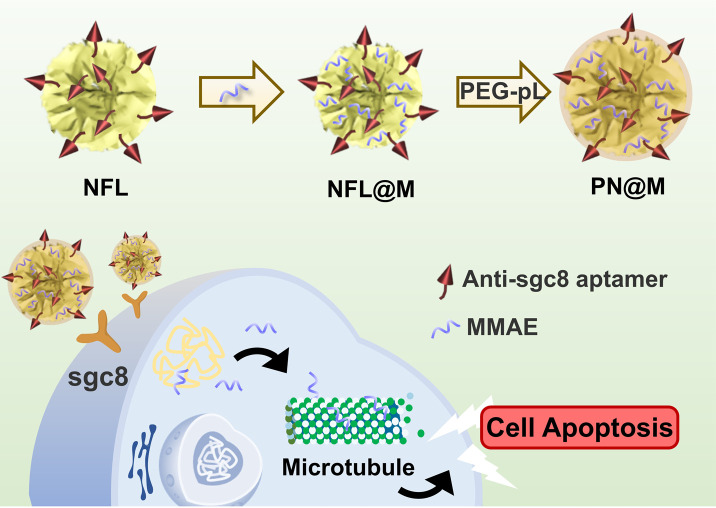
Schematic of the self-assembly polymer–DNA nanoflower for the targeted delivery of MMAE. MMAE: monomethyl auristatin E. PN@M: MMAE-loaded polymer–DNA nanoflower with anti-sgc8 aptamer modified. PEG–pL: abbreviation of PEG–pLysine.

In this study, we first report a targeted MMAE delivery system based on RCA-derived flower-like DNA structures (NFL) for resistant cancer therapy. We developed a delivery strategy for dolastatin-derived microtubule inhibitors loaded in the polymer–DNA assembled nanoflowers (PN) as shown in Fig. 1. The tumor-targeted aptamer (anti-sgc8 aptamer) and drugs were directly loaded through the synthesis of nanoflowers. In particular, MMAE was selected for loading owing to the relative regular size and high drug-loading efficiency of the MMAE-loaded nanoflower, namely, PN@M. With the guidance of the aptamer, the drug-loaded nanoflowers, PN@M, demonstrated enhanced accumulation in sgc8-positive MCF-7 cells and acted as effective tumor growth inhibition in a mouse model.

## Results and discussion

### Construction and characterization of the microtubule inhibitors-loaded nanoflower

Self-assembly DNA structure has been developed for effective drug delivery in many types of diseases.^37,42^ The nanoflowers used in this research were prepared based on the RCA according to previous reports.^36,38^ In a typical RCA reaction, DNA polymerase, assisted by a metal cofactor Mg^2+^, produces long-strand DNA containing repeated units from the designed circular DNA. Deoxyribonucleoside triphosphate (dNTP) is incorporated into the long-strand DNA to form the flower-like structure as a fuel. The circular DNA as the template is universal for customized incorporation of DNA functionalities. Based on the customized templates, nanoflowers can be constructed to have many advantageous properties, including simple DNA design and preparation, size tunability, and resistance to enzymatic degradation and denaturation.^36–38^ In this research, circular DNA containing the complementary sequence of anti-Sgc8 aptamer (ATCTAACTGCTGCGCCGCCGGGAAAATACTGTA) was used for the DNA template. The live-cell SELEX-developed anti-Sgc8 aptamer could recognize specifically and bind tightly to MCF-7 cells.^43,44^ The sequence of the template is shown in Table S2.† Efficient production of the long DNA strand spontaneously formed flower-like DNA structures and the incorporation of the PEG–pLysine was proved to have negligible influence on the reaction. Following the successful construction of the self-assembly nanoflower, the drug-loading performance of the polymer–DNA assembled nanoflower was optimized upon incorporation of PE, D-10, MMAF, and MMAE (Table S1 and Fig. S1†). The MMAE-loaded nanoflower, namely, PN@M, was chosen for further verification in anti-tumor therapy for the combination of excellent drug-loading efficacy (EE: 85.6 ± 5.0%) and uniform size distribution (diameter: 0.92 ± 0.29 μm). The microtubule inhibitor-loaded PN@M was supposed to exhibit enhanced anticancer activity due to the strategy of the targeted delivery.

**Fig. 2:**
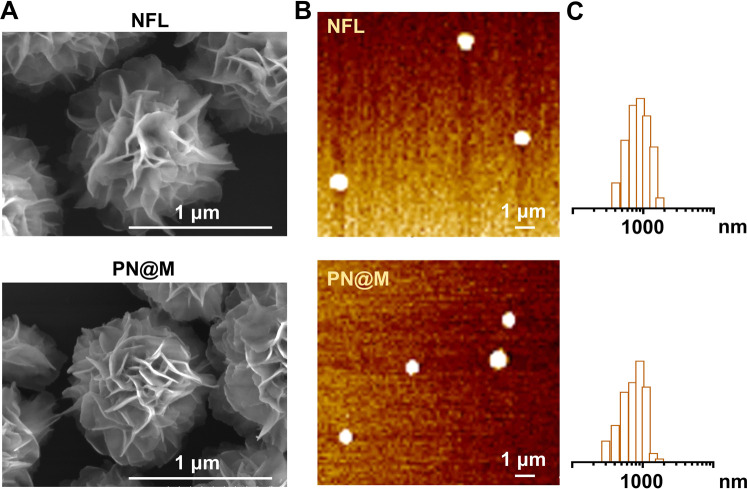
(A) SEM images of unloaded NFL and MMAE-loaded PN@M. Scale bar: 1 μm. (B) AFM images and size distribution of NFL and PN@M. The prepared samples (10 nM) were imaged with a MultiMode 8 AFM (Bruker) under the ScanAsyst-Fluid mode. Scale bar: 1 μm. (C) Size distribution analysis of NFL and PN@M.

Next, we characterized the drug carrier by scanning electron microscopy (SEM, Fig. 2A) and atomic force microscopy (AFM, Fig. 2B). Monodisperse assembled structures were detected and the average diameter of the drug-loaded PN@M (0.92 ± 0.29 nm) had no obvious difference with the unloaded NFL (1.12 ± 0.18 nm), as shown in Fig. 2C. The same results were collected in these results demonstrating that the loading of MMAE caused no adverse effects on the morphology of the DNA nanoflower. The SEM images of the NFL and PN@M indicated that there was no change in the flower-like shape after the modification. Taken together, this may be due to the optimized component in DNA nanoflower, which contains a relatively small number of PEG–pLysine. The drug-release behavior of the DNA nanoflower PN@M was subsequently investigated in PBS with physiological pH and the resultant cumulative release of MMAF *versus* time is shown in Fig. S2.† Note that 35.5% of the loaded MMAE was slowly released in PBS during the 48 h of the incubation. We concluded that the drug-release behavior of PN@M was a time-dependent manner and the sustained release performance is beneficial for drug enrichment in the tumor. As proven by previous research, drug leakage caused by sudden release could cause severe side effects during the admiration; however, there are several advantages of the sustained release including lesser frequency of administration, reduced side effects, and stable drug absorption levels in the blood and plasma.^45,46^ In summary, the DNA nanoflower with excellent drug-loaded performance holds great potential in drug delivery research.

### Cellular internalization analysis in cancer cells

**Fig. 3:**
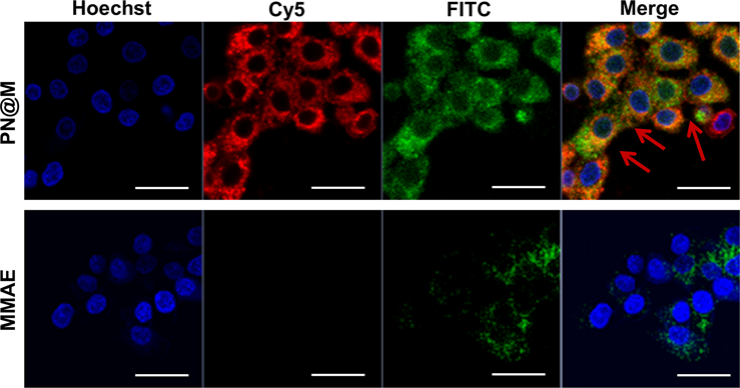
Confocal images of MCF-7 cells treated with different drugs. The drug concentration was based on 10 ng per mL FITC–MMAE. Nanoflower was labeled with Cy5, red; MMAE was labeled with FITC, green. The nucleus was stained with Hoechst, blue. Scale bar: 50 μm.

**Fig. 4:**
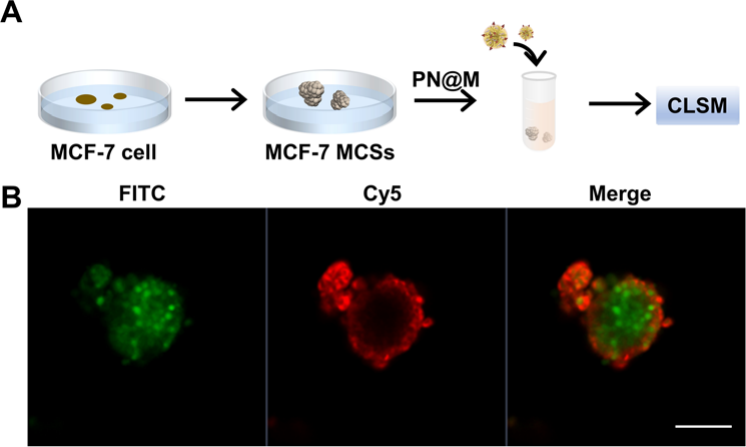
(A) Illustration of the preparation of the MCS internalization test. (B) Confocal images of MCF-7 MCSs. The drug concentration was based on 10 ng per mL FITC–MMAE. After incubating for 3 h, the spheroids were observed by CLSM in the confocal dish. Nanoflowers were labeled with Cy5, red; MMAE was labeled with FITC, green. Scale bar: 10 mm.

The intracellular delivery of MMAE into MCF-7 cells was tested in this study, which was proved to be Sgc8 positive.^47,48^ For fluorescence imaging, the DNA nanoflower was labeled with the fluorophore Cy5 (*λ*_ex_ = 650 nm, *λ*_em_ = 670 nm; red) on the DNA strand and the loaded MMAE was labeled with the fluorophore fluorescein isothiocyanate (FITC, *λ*_ex_ = 492 nm, *λ*_em_ = 518 nm; green). The dual-fluorophore-labeled drug delivery system was then incubated with MCF-7 cells and intracellular uptake of each component was independently monitored using confocal laser scanning fluorescence microscopy (CLSM) as shown in Fig. 3 and S3.† The enhanced signal was tested in PN@M treatment. The CLSM visualization clearly demonstrated the internalization of nanoflower and the accumulation of MMAE was significantly improved through aptamer modification. About 7.8 times higher MMAE accumulation in cells was measured by quantitative statistics. The same trend was observed in multicellular tumor spheroid (MCS) of MCF-7 cells (Fig. 4). MCSs are a kind of ideal 3D model for the investigation of drug penetration *in vitro* due to the multilayers of tumor cells and can resemble avascular micrometastases and intervascular regions of solid tumors.^49^ In our research, high accumulation and permeability of MMAE in MCS was achieved through PN@M treatment as expected. The result of the cellular internalization is in coincidence with our previous investigations. The fluorescence signal of FITC-labeled MMAE penetrated throughout the whole structure of the MCS. Interestingly, although the nanoflower-based delivery system increases the whole size of the therapeutic agents, the loaded MMAE can still penetrate MCS efficiently. This is probably because the flexible structure of the nanoflower and the modification of the targeted aptamer are critical in enhancing drug accumulation in cancer therapy.^50,51^ Similar to antibodies, recognition properties of aptamers are specific and could induce interactions of the ligands related to cell endocytosis and transport. The red signal represents DNA nanoflowers that did not fulfil all cells. Only cells on the edge of the MCS hold red fluorescence, which indicates effective drug release is vital for the nanoflower-based dolastatin-derived microtubule inhibitors delivery system. Therefore, the targeted microtubule inhibitors-loaded polymer–DNA assembled nanoflower PN@M holds great promise for enhanced therapeutic efficacy, given the optimal drug load performance.

### *In vitro* cell viability analysis

**Fig. 5:**
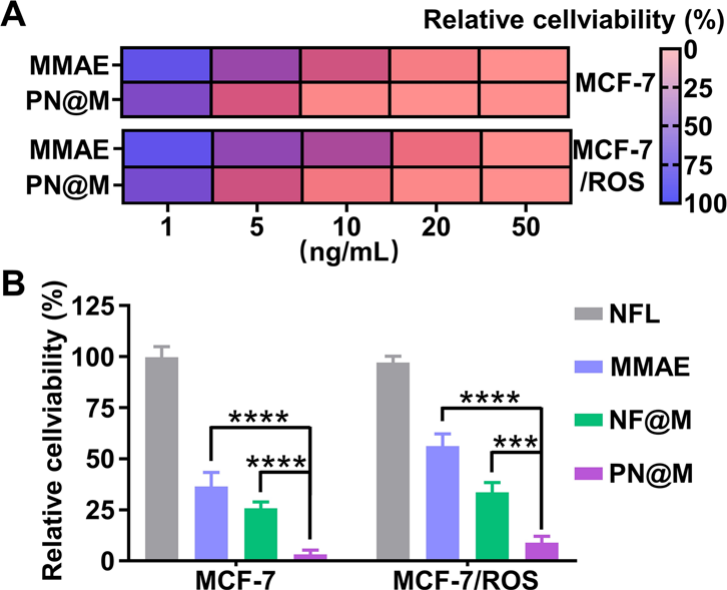
(A) Relative cell viability of MCF-7 cells and drug-resistant MCF-7/ROS cells treated with different concentrations of drugs. (B) Cell viability analysis of cells after the indicated treatments. NF@M:PN@M without aptamer. (***P < 0.001; ****P < 0.0001).

Encouraged by the excellent delivery performance of PN@M, the cellular cytotoxicity of PN@M in MCF-7 cells and resistant MCF-7/ROS cells was evaluated and compared between different treatments. During the past few decades, cancer resistance to chemical drugs has become a major problem for cancer treatment. As shown in Fig. 5A, PN@M exhibited significantly stronger cytotoxicity than free MMAE in both MCF-7 cells and resistant MCF-7/ROS cells. After we tested the cell viability of different components on the cell, the result indicated that PEG–pLysine is crucial in the enhancement of drug efficacy (Fig. 5B). The effective inhibitory in resistant cells was proved by cell apoptosis imaging (Fig. S4†). Based on the above results, the targeted MMAE delivery system has promising potential in cancer therapy.

### *In vivo* anti-tumor effect analysis

**Fig. 6:**
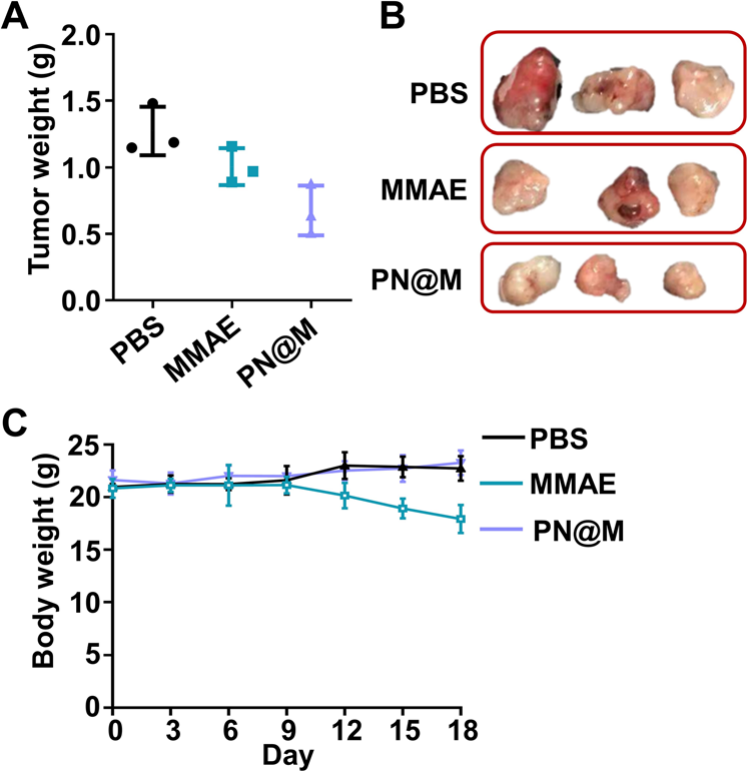
(A) Tumor weight of each group after the treatment of PBS, MMAE, and PN@M. The tumor-bearing mice were administered through intratumoral injections on day 0, day 3, and day 6 with a dosage of 1.0 mg kg^−1^ based on MMAE. (B) Pictures of the tumor harvested after the treatment. (C) Body weight changes of the mice during the treatment. The body weight was recorded every 2 days.

We further demonstrated the antitumor activity of PN@M in an MCF-7/ROS mouse xenograft tumor model. The mice were divided into three groups for anti-tumor efficacy studies. When the tumor reached 50 mm^3^, the mice were administered intratumoral injections on day 0, day 3, and day 6 with a dosage of 1.0 mg kg^−1^ based on MMAE. Changes in the tumor volume and body weight were monitored during the treatment. The treatment of PN@M exhibited an apparent reduction in the tumor weight and volume compared to free MMAE, as shown in Fig. 6A and S5.† While there was a slight increase in the tumor size in 12 days, the average tumor size in PN@M increased much more slowly than in the PBS and free MMAE group during the treatment. The smallest tumor size and weight were achieved through PN@M administration. The combination of the targeted aptamer introduction and PEG–pLysine modification showed the obvious tumor inhibitory effects towards drug-resistant cancer.

Under the given experimental doses in our research, the MMAE only showed a limited inhibitory effect on tumor growth as shown in tumor volume changes and tumor weight. Besides, weight loss occurred during the treatment of free MMAE (Fig. 6B). In contrast, negligible influence on the body weight of mice was detected in PN@M treatment. These results confirmed that the MMAE has limitations as a drug itself due to the non-specificity-caused systemic toxicity. The systematic safety of MMAE is broadly focused on since this highly toxic drug has repeatedly induced serious adverse events after a single administration at dose equivalents that are relatively greater than 1.0 mg kg^−1^.^7,13,52^ The strategy of loading MMAE by self-assembly of DNA nanoflower reduced the system toxicity during the treatment. The MMAE was loaded in the delivery system and based on the drug release curve in Fig. S2.† We believe that the sustained release behavior of the nanoflower is critical in the improvement of the biosafety in MMAE administration. The *in vivo* results proved that the PN@M can improve the therapeutic efficacy in the use of non-targeted chemotherapeutic drugs.

## Materials and methods

### Materials

MMAE was purchased from Abcam. PE, D-10, MMAF, and PEG–pLysine were purchased from Shanghai Aladdin Biochemical Technology Co., Ltd. Dulbecco's modified Eagle's medium (DMEM), and fetal bovine serum were purchased from Gibco. *Φ* 29 DNA polymerase (high concentration) and dNTPs were obtained from Enzymatics. 4% paraformaldehyde tissue fixative was obtained from Wuhan Servicebio Technology Co., Ltd. Annexin V/PI staining kit was purchased from Solarbio kit (Beijing, China). YeaRed (cat #10202; cat #40302) and Mycoplasma Removal Agent (cat #60703) were purchased from Yeasen, Shanghai, China. All the oligonucleotides and fluorophore-labeled oligonucleotides used in this work were obtained from Huzhou Hippo Biotechnology Co., Ltd. (Huzhou, China), and the oligonucleotides used in this work are listed in Table S2.†

### Preparation of the nanoflowers

To prepare the drug-loaded DNA nanoflowers, circularized templates (0.3 μM) were added with *Φ* 29 DNA polymerase (2 U μL^−1^), dNTPs (2 mM μL^−1^), and BSA in a buffer solution and microtubule inhibitors (1 mg mL^−1^) at 30 °C for 12 h. Then, the product was mixed with dolastatin-derived drugs (1 mg mL^−1^) and PEG–pLysine (5 kDa) at an N/P ratio of 0.1 : 1 to prepare polymer–DNA-assembled nanoflowers, namely, PNs. After the incubation at room temperature for 1 h, nanoflowers were washed with PBS and precipitated by centrifugation. The fluorophore-labeled DNA nanoflowers were synthesized using Cy5-labeled DNA strand (sequence: Cy5-ATCTAACTGCTGCGCCGCCGGGAAAATACTGTA) and fluorescein isothiocyanate modified MMAE. The morphologies of nanoflowers were observed by scanning electron microscopy (SEM, JEOL, JSM-7500F) and atomic force microscopy (AFM, MultiMode 8, Bruker). The drug loading efficacy was measured by quantifying MMAE through high-performance liquid chromatography or HPLC.

### Characterization of the nanoflowers

The freshly purified nanoflowers (100 nM, 10 μL) were deposited onto freshly cleaved mica and deposited for 10 min. The prepared samples were imaged with a MultiMode 8 AFM (Bruker) under a ScanAsyst-Fluid mode.

For SEM imaging, 10 μL of the sample was deposited and dried at room temperature. SEM imaging was performed using a Regulus 8100 (Hitachi Limited).

Dynamic light scattering was performed on a Malvern Zetasizer Nano-ZS (Malvern Instruments, U.K.). NFL and PN@M were dispersed in PBS buffer and the test was conducted at 25 °C.

### Drug release of the nanoflowers

The prepared nanoflowers were dispersed in PBS buffer at a pH of 7.4 and the mixture was sealed in a dialysis bag (10 kDa). The dialysis bag was immersed in 10 mL of PBS with the corresponding pH and then incubated by shaking (200 rpm min^−1^) at 37 °C. The released MMAE was separated using the Amicon-stirred cell (equipped with 5 kDa filter) at different time points (0.5 h, 1.0 h, 2.0 h, 8.0 h, 16.0 h, 24 h, and 48 h). HPLC was used for free MMAE quantification.

### High-performance liquid chromatography conditions

For quantification of the free MMAE, an Agilent 1100 HPLC system (equipped with an Agilent Hypersil ODS C18 HPLC column) was used to quantify the MMAE-containing samples. Samples containing MMAE were diluted in methanol and filtered before injection (0.22 μm). The HPLC conditions were as followed; constant 25% A (0.1% TFA in acetonitrile) and 75% B (0.1% TFA in ddH_2_O) for 3 min, linear gradient to 45% A from 3 min to 19 min, linear gradient to 95% A from 20 min to 22 min, constant 95% from 23 min to 25 min, linear gradient to 30% A from 26 min to 27 min, and constant 30% A from 27 min to 30 min with a flow rate of 0.8 mL min^−1^ at 220 nm.

### Cellular uptake assay

Human cancer cells MCF-7 cells (American Type Culture Collection) were seeded in 24-well plates (1 × 10^5^ cells per well) with DMEM medium containing 10% FBS and 1% penicillin/streptomycin. Cells were grown and maintained in a humidified atmosphere with 5% CO_2_ at 37 °C. After adhesion, cells were incubated with a nanoflower for 4 h. The drug concentration was based on 10 ng per mL FITC–MMAE. After incubation, the cells were washed with PBS three times and fixed in 4% paraformaldehyde for 15 min. Nuclei were counterstained with DAPI. The cells were imaged under a confocal laser scanning microscopy (CLSM, IX81; Olympus, Tokyo, Japan).

### Multicellular tumor spheroid internalization test

Multicellular tumor spheroid (MCS) was used for verifying the internalization of nanoflower. Note that 1 × 10^6^ MCF-7 cells were seeded in a flask and incubated for 72 h. The MCSs were then added with PN@M (10 ng per mL MMAE). After incubating for 3 h, the spheroids were collected and fixed with 4% PFA (w/v) at room temperature for 1 h. Finally, the spheroids were observed by CLSM.

### *In vitro* cytotoxicity study

Cell viability was tested through Annexin V/PI staining and CCK8 assay. MCF-7 cells were seeded in six-well plates and cultured for 24 h. Then, the medium was replaced with fresh DMEM containing 10% FBS and each component. The drug concentration was based on 50 ng per mL MMAE. After 6 h of incubation, cells were washed with PBS three times and fixed in 4% paraformaldehyde for 15 min. For Annexin V/PI staining, MCF-7 cells were treated with different drugs and further stained for 10 min. Then, cells were observed through CLSM.

For the CCK8 assay, MCF-7 cells were seeded in a 96-well plate with 10^4^ cells per well and cultured overnight for cell adhesion. The medium was replaced by 100 μL of DMEM medium containing different concentrations of drugs. The drug concentration ranged from 1 to 50 ng mL^−1^, based on MMAE. After 24 h, the mixture of the drug-containing medium was removed and the CCK8 solution was added and incubated for 1 h, and the absorbance was measured at 450 nm using the Spectra Max M5 microplate reader.

### *In vivo* anti-tumor study

All animals received care in compliance with the guidelines outlined in the Guide for the Care and Use of Laboratory Animals. The procedures were approved by the Institutional Animal Care and Use Committee of Hainan Medical University with ethics approval (HYLL-2023-182). To generate xenografts, 100 μL of 5 × 10^6^ cells in PBS were injected in the upper right blanks of the BALB/c nude (female, 5–6 weeks). When the tumor volume reached 50 mm^3^, the mice were split into three groups randomly and 25 μL of PBS, free MAEE, or PN@M were administrated by tail vein injection at days 0, 3, and 6. The tumor volume and weight were recorded every two days. At 18 days after tumor inoculation, tumors were collected and weighed.

### Statistics

All experiments were repeated at least three times and each condition was analyzed in triplicate. The statistical significance of differences between experimental and control groups was determined using a Student's *t*-test. Significant differences are denoted in the figures.

## Conclusions

In summary, to improve the drug effects of dolastatin-derived drugs, we designed a simple, highly drug-loaded, and specific aptamer-modified polymer–DNA-assembled nanoflower. Taking advantage of the unique features of the DNA nanoflower, this multifunctional nanoflower serves as a delivery system for dolastatin-derived microtubule inhibitors in cancer therapy. Recent advancements highlight the successful application of programmable self-assembly DNA nanoflowers in biosensing, bioimaging, and therapeutics. By virtue of DNA sequence customization, we integrated multiple functional nucleic acids within the individual DNA self-assembly nanoflowers. This approach addresses the limitations of dolastatin-derived microtubule inhibitors in cancer therapy. The targeted delivery system, PN@M, exhibits excellent bioavailability and therapeutic efficacy, allowing recognition, accumulation, and sustained release within breast cancer cells. This enhanced performance results in high cytotoxicity against both MCF-7 cells and drug-resistant MCF-7/ROS cells. Moreover, *in vivo* therapy results demonstrate that PN@M has targeting capabilities and can increase therapeutic efficacy. Given that drug resistance and toxicity are major obstacles in cancer treatment, this microtubule inhibitor delivery system holds significant clinical promise.

## Author contributions

TW: conceptualization, data curation, formal analysis, writing-original draft. YS: writing-review and editing, formal analysis, validation, software. TY: writing-review & editing. PZ: data curation, methodology. ZY: funding acquisition, supervision, writing, review and editing. BY: funding acquisition, supervision, investigation, writing, review and editing. All authors read and approved the final manuscript.

## Conflicts of interest

There are no conflicts to declare.

## Supplementary Material

RA-014-D3RA08146J-s001
