# Supplementary material for: Polymer–DNA assembled nanoflower for targeted delivery of dolastatin-derived microtubule inhibitors
Source: RSC Adv. 2024 Mar 21;14(14):9602–8. doi: 10.1039/d3ra08146j (PMC10956646; doi:10.1039/d3ra08146j)
Supplement: RA-014-D3RA08146J-s001 [file RA-014-D3RA08146J-s001.pdf]

Electronic Supplementary Material (ESI) for RSC Advances.
This journal is © The Royal Society of Chemistry 2024

Polymer-DNA assembled Nanoflower for Targeted Delivery of Dolastatin-Derivatived Microtubule Inhibitors

Tiantian Wu, [§a, b] Yanqiang Shi, [§ a] Tao Yang, [b] Pengxuan Zhao, [*b] Zhu Yang, [*c,d] and Bin Yang [*a]

a. Molecular Diagnosis and Treatment Center for Infectious Diseases. Dermatology Hospital. Southern Medical University. Guangzhou 510091, China.

b. Department of Neurosurgery, Hainan General Hospital/Hainan Affiliated Hospital of Hainan Medical University, Haikou, 570311, China.

c. Department of Neurosurgery, Neurosurgery Research Institute, the First Affiliated Hospital of Fujian Medical University, Fuzhou, 350005, China.

d. Department of Neurosurgery, National Regional Medical Center, Binhai Campus of the First Affiliated Hospital of Fujian Medical University, Fuzhou, 350212, China.

Supplementary Data

Table S1. Drug loading performance of the nanoflower.

Name of the nanoflower	Drug (MW)	LC[1] (μg/mL)	EE[2]%	Diameter (μm)
PN@PE	701.98	433 ± 19	86.5 ± 3.8	1.74 ± 0.58
PN@D-10	785.09	407 ± 23	81.3 ± 4.6	1.33 ± 0.31
PN@MMAE	731.96	351 ± 34	75.1 ± 6.8	1.16 ± 0.48
PN@MMAE (PN@M)	717.98	426 ± 25	85.6 ± 5.0	0.92 ± 0.29

Table S2. DNA sequences of the template.

Name	Sequence (5'-3')
**Template**	Phosphate-<br>CATATCCCTAGGGATATGTCTAACCGTACAGTATTTCCCGGCGG<br>CGCAGCAGTTAGATTTGTTGGTACGTTAATACGACT
**Anti-Sgc8 aptamer**	ATCTAACTGCTGCGCCGCCGGGAAAATACTGTA
**Cy5-aptaemr**	Cy5-ATCTAACTGCTGCGCCGCCGGGAAAATACTGTA

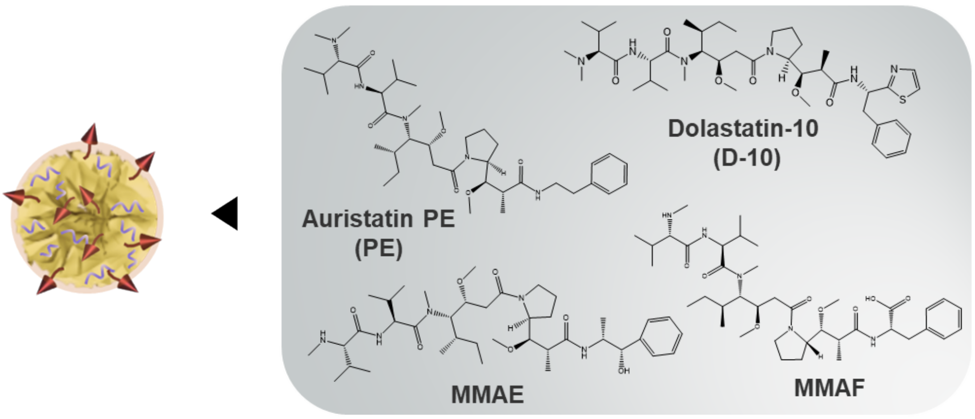

Figure 1. Illustration of the drug-loaded nanoflower.

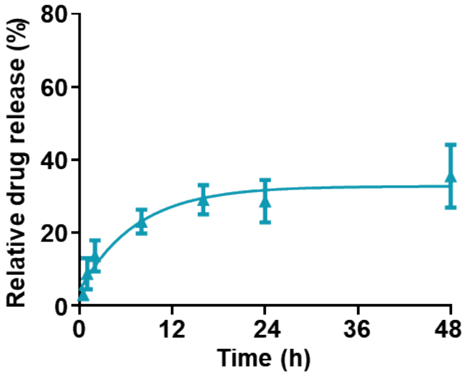

Figure 2. Release of MMAE in PBS at 37 °C.

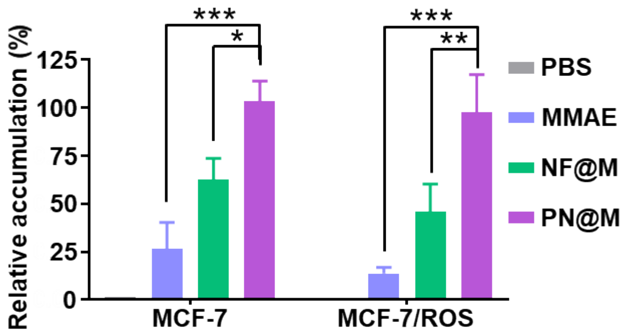

Figure 3. Relative MMAE accumulation in cells. The accumulation efficacy was measured by quantifying MMAE through HPLC. (** P < 0.01; *** P < 0.001; **** P < 0.0001).

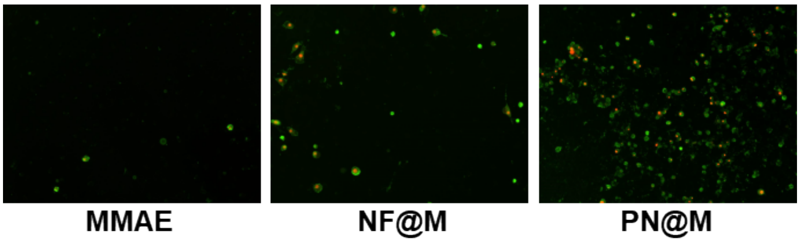

Figure 4. Cell apoptosis imaging. MCF-7/ROS cells were stained with Annexin V-FITC and PI after being cultured with different drugs.

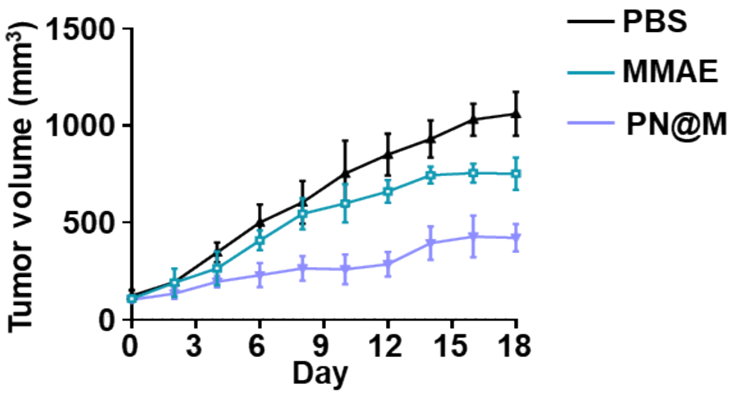

Figure 4. Tumor volume changes during the treatment of different drugs.
